# Machine Learning and SHapley Additive exPlanation-Based Interpretation for Predicting Mastitis in Dairy Cows

**DOI:** 10.3390/ani16020204

**Published:** 2026-01-09

**Authors:** Xiaojing Zhou, Yongli Qu, Chuang Xu, Hao Wang, Di Lang, Bin Jia, Nan Jiang

**Affiliations:** 1 Department of Information and Computing Science, Heilongjiang Bayi Agricultural University, No. 5 Xinyang Road, Daqing 163319, China; zhouxiaojing7924@126.com (X.Z.); langdi001@126.com (D.L.); jiangnan2226520@163.com (N.J.); 2 The Key Laboratory of Green and Low-Carbon Agriculture in the Northeast Plain, Ministry of Agriculture and Rural Affairs, Heilongjiang Bayi Agricultural University, No. 5 Xinyang Road, Daqing 163319, China; 3 National Key Laboratory of Veterinary Public Health and Safety, College of Veterinary Medicine, China Agricultural University, 2 Yuanmingyuan West Road, Beijing 100193, China; xuchuang7175@163.com; 4 Animal Husbandry and Veterinary Branch of Heilongjiang Academy of Agricultural Science, Qiqihar 161005, China; tlwanghao777@126.com (H.W.); wenxi311@126.com (B.J.)

**Keywords:** mastitis prediction, milk yield, milk electrical conductivity, quantile regression, machine learning, SHAP

## Abstract

SHapley Additive exPlanations (SHAP) analysis has been applied in disease diagnosis and treatment effect evaluation. However, its application in the prediction and diagnosis of dairy cow diseases remains limited. We investigated whether the variance and autocorrelation of deviations in daily activity, rumination time, and milk electrical conductivity, along with daily milk yield, could be used to predict clinical mastitis in dairy cows using popular machine learning (ML) algorithms and identifying key predictive features using SHAP analysis. Quantile regression (QR) with second- or third-order polynomial models with the median or upper quantiles was used to process raw data from mastitic and healthy cows. Nine variables from the 14-day period preceding mastitis onset were identified as significantly associated with mastitis through logistic regression. These variables were used to train and validate prediction models using eleven classical ML algorithms. Among them, the partial least squares model demonstrated superior performance, achieving an AUC of 0.789, sensitivity of 0.500, specificity of 0.947, accuracy of 0.793, precision of 0.833, and F1-score of 0.625. SHAP analysis results revealed positive contributions of three features to mastitis prediction, whereas two features had negative contributions. These findings provide a theoretical basis for developing clinical decision-support tools in commercial farming settings.

## Simple Summary

Mastitis in dairy cows causes major economic losses, reduces milk quality, and increases environmental burden from antibiotic use. This study demonstrated that farm sensor data processed via quantile regression combined with machine learning and SHapley Additive exPlanations analysis can be used to detect early, subtle signs of mastitis before clinical symptoms appear. Such early warning systems could help farmers to act sooner, improving cow health, reducing treatment costs, and minimizing antibiotic use. These findings support the move toward smarter and more sustainable dairy farming practices, with benefits for farmers, consumers, and the environment.

## 1. Introduction

Mastitis is one of the most common diseases in dairy cows, negatively impacting animal welfare, the dairy industry’s economy, the environment, and public health. Over the past decade, with the rapid development and widespread application of artificial intelligence in animal husbandry, numerous efforts have been made to develop mastitis prediction and detection methods using multi-dimensional real-time data from production, management, and environmental sources. In recent years, machine learning (ML) algorithms have been widely used across various domains, including disease prediction, diagnosis, and classification [1,2,3]. ML algorithms were employed to develop models predicting subclinical mastitis using monthly routine milk recording and climatic data [4], detecting clinical mastitis using quarterly time-series milking data [5]. ML-based models identified early metritis in postpartum dairy cows using physiological, behavioral, and milk quality parameters [6]. To predict dairy cows’ core body temperature, ML models were built and SHapley Additive exPlanations (SHAP) analysis was applied to boost both accuracy and interpretability for practical livestock management [7]. Quantile regression (QR) has gained popularity for its robustness to outliers, adaptability to heteroscedasticity, and flexibility in modeling non-normal data distributions, making it well-suited to capturing complex variable relationships. A fourth-order QR model was recommended to reduce the sensitivity of lactation curves to milk yield fluctuations [8]. To derive resilience indicators from deviations, individual lactation curves were similarly modeled using QR and examined resilience indicators’ genetic determinants and associations with milk yield and longevity [9]. A quantile regression (0.6 quantile) was applied to estimate total expected milk production and overall milk loss in dairy cows [10]. Individual lactation curves were fitted using polynomial QR (0.5 quantile) to derive expected lactation curves before investigating dairy cow resilience of herds from U.S. dairy farms [11]. SHAP analysis, a powerful model interpretation tool grounded in Shapley values from game theory, quantifies the contribution of individual features to model predictions. It has been increasingly applied in disease diagnosis and treatment effect evaluation [12,13,14]. However, its application in the prediction and diagnosis of dairy cow diseases remains limited.

To identify and characterize patterns in high-volume real-time data, assuming minor deviations in multidimensional variables can be indicative of mastitis onset, we first applied QR to preprocess data over four time windows before diagnosis, using second- and third-order polynomial models with median (0.5) and upper (0.75) quantiles. Subsequently, 11 ML algorithms were employed to build predictive models, evaluated via 6 performance metrics; SHAP analysis was then applied to interpret feature contributions, along with biological explanations.

## 2. Materials and Methods

The QR method adopted in the data processing stage required specification of the time window (days before mastitis onset) and the polynomial order for regression analysis. In this study, the variable “days” was set at 28, 21, 14, and 7 days before mastitis onset. Parity, days in milk (DIM), and season were considered to assess differences in forecast performance. To obtain reliable results and provide better decision-making support for farm managers, data from healthy cows with similar DIM, daily milk yield, and parity as mastitic cows were also collected.

### 2.1. Animals, Housing, and Feeding

We collected original data on Holstein lactating cows between January 2023 and May 2024 from a commercial farm (with an average of 650 lactating cows and a total stock of 1300 cows per year) in Northeast China, which serves as the practice base of the Heilongjiang Bayi Agricultural University. The farm is located at 48.10–48.21° N and 126.35–126.47° E. The region has a cold temperate, continental monsoon climate, with an annual mean temperature ranging from −5 °C to 5 °C.

All lactating cows were housed in uniform pens with identical characteristics: enclosed barns with solid concrete floors and sawdust bedding, accommodating 60–100 cows per pen with equivalent cubicles, feeding and watering areas, and feed bunks. The farms’ attendants cleaned the cattle pens and resting beds several times every day. The barns were naturally ventilated and equipped with sprayers at 20 m intervals during summer. In winter, hot-water pipes were installed to alleviate cold stress. Cattle are dewormed annually in spring and autumn via subcutaneous injection of acetylamino abamectin. In addition, each cow was injected 1 mL foot and mouth disease bivalent (type O and A) vaccine, inactivated (Strain O/MYA98/BY/2010 + Strain RE-A/WH/09, JinyuBaoling Co., Ltd., Hohhot, China) three times every year, in April, August and December, respectively.

All cows were equipped with neck collars to record physical activity and rumination time, which were recorded with the HR-Tag monitoring system (SCR Engineers Ltd., Netanya, Israel), averaged at 2 h intervals, and stored over 24 h. Cows were fed a total mixed ration (TMR) twice daily (0730 h–0810 h and 1330 h–1410 h), and feed intake was monitored daily. The feed was routinely repositioned toward the feed bunk as needed, and the cows had ad libitum access to fresh water. Before calving, cows received a prepartum TMR with a forage-to-concentrate ratio of 65:35 on a dry matter (DM) basis, which was adjusted to 55:45 postpartum. Primiparous and multiparous cows were fed the same TMR from 1 DIM until the end of lactation.

Milking occurred four times daily (0000, 0600, 1200, and 1800 h) using two 32-stall parallel milking parlors (SCR Engineers Ltd., Netanya, Israel, 2015). All performance data were automatically transferred during each milking to the herd management software (DataFlow II, SCR Engineers Ltd.), and daily milk yield was calculated as the total milk of all cow quarters collected per cow per day. Additional information on parity, DIM, disease detection and diagnosis, and reproductive events was recorded using the Foidn dairy farm intelligent management system (Foidn I, Nanjing Fengdon Technology Co., Ltd., Nanjing, China, 2016).

### 2.2. Data Collection and Study Design

During the experimental period, data were collected from both mastitic and healthy cows, including daily milk yield, physical activity, rumination time, and milk electrical conductivity. Data editing was conducted using the following exclusion criteria: parameters monitored by the automated monitoring system with missing data; cows lacking complete records for ≥35 days prior to diagnosis; and cows moved between herds more than twice during the period of data collection. For the healthy group, cows were matched to mastitic cows by parity and DIM, and the reference day (d0) was defined to correspond to the diagnosis day of the mastitic group. Cows were checked daily by farm staff for signs of disease or injury, reproductive status, and survival, as well as the warning events provided by the automated monitoring system. Health assessments were routinely performed during the transition period; the entire herd underwent hoof trimming by the farm-employed trimmers once in spring and once in autumn. Clinical signs of mastitis were regularly examined by observing the udder and milk (i.e., hard quarters, heat or swelling, clots, flakes, lumps, or clear/yellow milk) from calving to day 28, and every 3 days thereafter throughout lactation. Once a cow is diagnosed with mastitis, to prevent cross-infection, it should be immediately moved from its current barn to a dedicated barn for treatment. Time from detection to diagnosis should not exceed 6 h, and the cow’s basic information, date of diagnosis, and staff who detected and diagnosed the diseases, among other information, were entered into the management system software within 5 min after diagnosis according to the standard operating procedures. The current experiment only considered cows with a single mastitis episode within each time window, nor were other diseases taken into account. The final dataset comprised 255,772 records from 68 mastitic cows and 154 healthy controls, with 12 raw variables, including parity, season of mastitis occurrence, DIM, daily milk yield, activity, 3 rumination-related variables, and 4 milk electrical conductivity variables (Table 1). Before data processed by quantile regression, the data was split into training subset and the test one, that is, the 70% subset of the observations served as training data for model construction, and the remaining 30% as test data to assess model performance, thus, there are 48 mastitic cows and 108 healthy ones in the training data set, 20 positive and 46 negative ones in the test data set, respectively.

### 2.3. Data Preprocessing

To analyze the fluctuations in the variables 28 days before mastitis onset, including milk yield, activity, rumination time, and milk electrical conductivity, differences between raw and predicted values were assessed with R version 4.5.1 (R Core Team, 2025, Auckland, New Zealand. Available online: https://www.r-project.org/ (accessed on 2 July 2025)), using a second/third-order polynomial QR at the 0.5/0.75 quantile by the quantreg package, as described by Poppe et al. [15]. Quantile residuals were defined as daily deviations of the original values from the fitted values. More specifically, residuals were calculated for each variable, followed by the computation of logarithmic variance. This process involved obtaining absolute residuals, applying a logarithmic transformation, and calculating the variance of the transformed residuals, yielding the log-transformed variance of deviations (lnVAR). In addition, autoregression was performed for each variable and the lag-1 autocorrelation fitting values (acf) from the daily raw data. Furthermore, data for the same variables at 21, 14, and 7 days before mastitis onset were preprocessed using second/third-order polynomial QR at the 0.5/0.75 quantile to derive the lnVAR and acf of the nine variables.

### 2.4. Logistic Regression Analysis

Univariate and multivariate logistic regression analyses were employed to analyze the lnVAR and acf for the variables from the QR of the raw data to screen for significant factors associated with mastitis in dairy cows.

### 2.5. Machine Learning Algorithms

Before data processing by QR, the data involved in the experiment were randomly divided into training set and test sets by the dependent variable “Species” (binary: “0” for “healthy cows” vs. “1” for “clinical mastitis cows”) using the “caret::createDataPartition()” function in R 4.5.1. Moreover, to ensure result repeatability, “set.seed()” was adopted for these algorithms. Considering the size of the sample used for modeling, and the comparisons as illustrative rather than exhaustive, the hyperparameter settings for some machine learning algorithms were set to a fixed “k”.

For the statistically significant variables selected in the logistic regression, 11 ML algorithms under the “mlr3” framework of the R software were employed to construct prediction models on cow mastitis. Specifically, logistic regression analysis was performed using the GLM model in the “glm” package with “family” set to “binomial.” Random forest (RF) is an ensemble learning method based on decision trees that constructs multiple trees and combines their outputs through majority voting. It is valued for its resistance to overfitting and ability to evaluate feature importance. The “randomForest” package was used with “ntree” set to 100. The support vector machine (SVM) with a radial basis function (RBF) kernel maps data into a high-dimensional space using the kernel trick, enabling complex classification boundaries and rendering it suitable for small to medium-sized datasets. The “SVM” package was applied with “kernel” = “radial”. Gradient boosting, a general implementation of adaptive boosting, was employed to handle complex data structures and loss functions. The “mfinal” package was used with “mfinal” = 50. Decision tree (DT) models were implemented with the “rpart” package (method = “class”, “cp” = 0.01, “minsplit” = 5). Naive Bayes (NB), a probabilistic classifier based on Bayes’ theorem, was constructed using the “naivebayes” package. K-nearest neighbors (KNN), a lazy learning algorithm that classifies samples based on the majority vote of their nearest neighbors, was applied using the “kknn” package with “k” = 5 and “distance” = 2. Partial least squares (PLS), a regression-based dimensionality reduction method that improves stability and interpretability when predictors are highly correlated, was implemented using the “plsr” package with “ncomp” = 2. Adaptive boosting (Adaboost), a quintessential algorithm within the boosting family, iteratively modifies sample weights, emphasizing difficult-to-classify samples and enhancing the overall performance of weak classifiers. The “gbm” package was used with distribution = “bernoulli,” “n.trees” = 50, “interaction.depth” = 2, “shrinkage” = 0.01, “n.minobsinnode” = 1, and “bag.fraction” = 0.5. The neural network (NN) emulates the connections between neurons in the human brain, automatically learning abstract feature representations of data through hidden layers. The “nnet” package was applied with size = 3, “maxit” = 500, and “trace” = FALSE. Linear discriminant analysis (LDA) seeks projection directions that maximize inter-class discrimination, commonly utilized for classification and dimensionality compression; herein, the “lda” package was selected.

Accounting for the imbalance in the output class and to comprehensively assess the model performance and identify the optimal model across different time periods (28, 21, 14, and 7 days before diagnosis), polynomial orders (second or third), and quantiles (0.5 or 0.75), we adopted sensitivity, specificity, accuracy, precision, F1-score and area under the receiver operating characteristic (ROC) curve (AUC) value (95% confidence interval) as evaluation metrics, which were defined as follows:

Sensitivity = TP/(TP + FN),(1)

Specificity = TN/(TN + FP),(2)

Accuracy = (TP + TN)/(TP + TN + FP + FN),(3)

Precision = TP/(TP + FP),(4)

F1-score = 2 × Precision × Sensitivity/(Precision + Sensitivity),(5)

where TP, FN, TN, and FP are true positives (the number of cows with clinical mastitis, predicted as clinical mastitis cows), false negatives (the number of cows with clinical mastitis, predicted as healthy cows), true negatives (the number of healthy cows, predicted as healthy cows), and false positives (the number of healthy cows, predicted as with clinical mastitis cows), respectively. The AUC value is used to measure the classification performance of a model. The closer the value is to 1, the better the performance of the model. The ROC was plotted by using the “pROC” package in R, with the x-axis expressing 1-specificity and the y-axis depicting sensitivity. The evaluating metrics of sensitivity, specificity, accuracy, precision, and F1-score of each model were calculated using the “confusionMatrix” of the R software 4.5.1.

### 2.6. SHAP Visualization

SHAP is a game-theory-based model explanation approach that measures the contribution of each feature to ML model predictions. The primary merits of SHAP analysis are its high interpretability, distinctly identifying the features driving model decisions; visualized results, facilitating an intuitive comprehension of the decision-making process of the model through global and local interpretability visualizations (global visualizations encompass feature importance plots, beeswarm plots, and dependence plots, whereas local visualizations entail waterfall plots and force plots); and broad applicability, making it suitable for various ML models. After constructing the diagnostic model, the SHAP method was used to interpret the model. By calculating the Shapley value of each feature, one can readily discern its positive or negative influence on model predictions, helping to unveil how and to what extent key factors affect mastitis. In the present study, the package “shapviz” in R software 4.5.1 was used to conduct SHAP analysis.

### 2.7. Statistical Analyses

The aforementioned variables were statistically analyzed unless otherwise stated. After removing missing data and outliers, descriptive statistics were performed to characterize the measures of location and variability by means of frequency distribution tables and histograms. Thereafter, the χ^2^ tests and *t*-test were performed for categorical outcomes and continuous variables, respectively. For *t*-tests, normality of data distribution was assessed using the Shapiro–Wilk test. Variance homogeneity was verified via Levene’s test. *p*-values < 0.05 denoted statistical significance (trends declared at 0.05 < *p* ≤ 0.10). For between-group comparisons of each variable, “aov” was used in R software 4.5.1, and “Tukey HSD” for post-hoc tests.

## 3. Results

The approximate prevalence of clinical mastitis in the commercial dairy farms ranged from 8% to 30% per 365 days during the experimental period. A total of 255,772 records from 68 mastitic and 154 healthy cows with similar parity, lactation stage, and milk yield were included in the experiment (Table 1). Parity for mastitis and healthy cows were 3.43 ± 1.27 and 3.03 ± 1.68, with age (months) of 51.87 ± 13.19 and 49.32 ± 26.73, respectively. DIM for mastitis and healthy cows were 136.91 ± 73.67 and 129.34 ± 51.57. Peak milk yield (PMY) and DIM at PMY in mastitic and healthy cows were calculated. The PMY of mastitic cows was 6.96 ± 2.16 (kg/day) higher than that of the healthy cows (*p* < 0.01), with values of 65.24 ± 7.28 vs. 58.27 ± 10.61; the DIM at PMY was 55.33 ± 24.61 (day) for mastitic cows and 58.31 ± 20.65 (day) for healthy cows, with no statistically significant difference between them. These findings support that high-yielding cattle are prone to mastitis.

### 3.1. lnVAR and Acf for the Variables from the QR of the Raw Data

**Figure 1:**
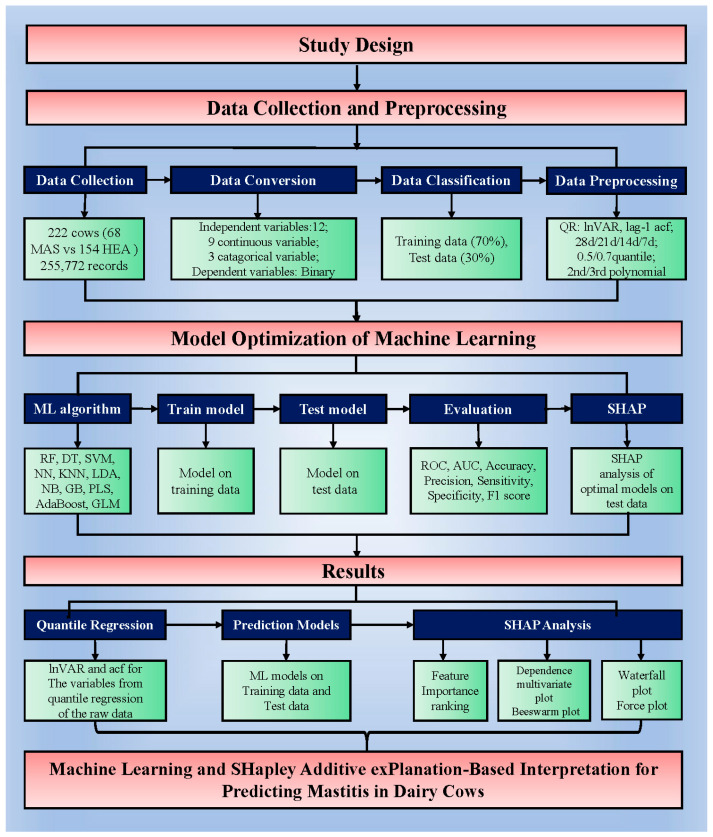
Flowchart of the study design. Abbreviations: MAS: mastitis; HEA: healthy; lnVAR: the log-transformed variance of deviations by quantile regression for each variable; acf: the lag-1 auto-correlation fitting values derived from autoregression; RF: random forest; DT: decision tree; SVM: support vector machine; NN: neural network; KNN: k-nearest neighbors; LDA: linear discriminant analysis; NB: naive bayes; GB: gradient boost; PLS: partial least squares; AdaBoost: AdaBoost; GLM: logistic regression analysis; ROC: receiver operating characteristic curve; AUC: area under ROC; SHAP: SHapley Additive exPlanations.

Nine variables, including daily milk yield from parallel milking system, daily activity, daily rumination time, rumination deviation per 2 h, sum of absolute values of the weighted rumination variation, peak electrical conductivity of milk, daily percentage change in the electrical conductivity of milk, standard deviation change in conductivity, and standard deviation of maximum conductivity change in the last three shifts, from the auto-monitoring system were preliminarily processed before QR. Then, considering “days before mastitis onset” as a reference, QR was performed for four time periods (28, 21, 14, and 7 days before pre-onset) with the polynomial transformation orders set as second and third orders, and the “tau” (quantile) parameter set as 0.5 and 0.75 in the R program (R 4.5.1, https://www.r-project.org (accessed on 2 July 2025)). Consequently, 16 datasets were generated for analysis in the ML algorithms, each containing 21 variables: 9 variables related to lnVAR and 9 related to acf for the variables from the QR of the original data, coupled with categorical variables, including parity, lactation stage, and onset season. Next, lnVAR and acf were used in logistic analysis to screen for significant variables related to mastitis in dairy cows. A flowchart of the study design is shown in Figure 1.

### 3.2. Variables Screened by Logistic Regression Analysis

As the dependent variable was a binary categorical variable, univariate and multivariate logistic regression analyses were used to screen for significant variables correlated with dairy cow mastitis. For the data from 14 days before mastitis onset (a third-order polynomial quantile regression at the 0.75 quantile), nine key features were identified at a significance level of *p* < 0.05, with no collinearity. Comparisons of the lnVAR and acf features identified by binary logistic regression based on QR for this group are presented in Table 2. Results of the other three time periods before mastitis onset, obtained using second/third-order polynomial QR at the 0.5/0.75 quantiles, are presented in Supplementary Table S1.

### 3.3. Prediction Models Built by ML Algorithms

**Figure 2:**
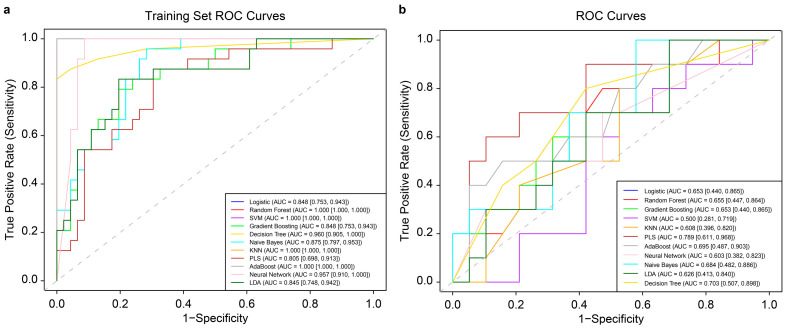
Receiver operating characteristic (ROC) curves of 11 machine learning (ML) models trained on 14-day data before mastitis onset, processed with third-order polynomial quantile regression at the 0.75 quantile. (**a**): ROC curve of the 11 ML models on the training data, with 95% confidence intervals of ROC-AUC from cross-validation shown in the bottom right corner. (**b**): ROC curves of the same 11 ML models on the corresponding test data, with 95% confidence intervals of ROC-AUC likewise indicated. SVM: support vector machine; KNN: k-nearest neighbors; PLS: partial least squares; LDA: linear discriminant analysis.

In this study, 11 ML algorithms were adopted to develop mastitis prediction models. After optimization with 5-fold cross-validation, the PLS model based on 14 days of data from a third-order polynomial QR at the 0.75 quantile demonstrated the best performance in internal validation. For the test set, the model achieved a sensitivity of 0.500, specificity of 0.947, accuracy of 0.793, precision of 0.833, F1_Score of 0.625, and AUC of 0.789 (95% confidence interval (CI): 0.611–0.968). Figure 2 shows the ROC curves of the models on training and test data, with AUC values and CIs. The classification threshold was determined using the Youden index (sensitivity + specificity − 1). ROC curves for the 11 ML models trained on 7-, 21-, and 28-day datasets, processed with second/third-order polynomial QR at the 0.5/0.75 quantile, are presented in Supplementary Figures S1–S3. The evaluation metrics of the optimal ML model for test data from these four time windows are summarized in Table 3.

### 3.4. SHAP Visualization Analysis

**Figure 3:**
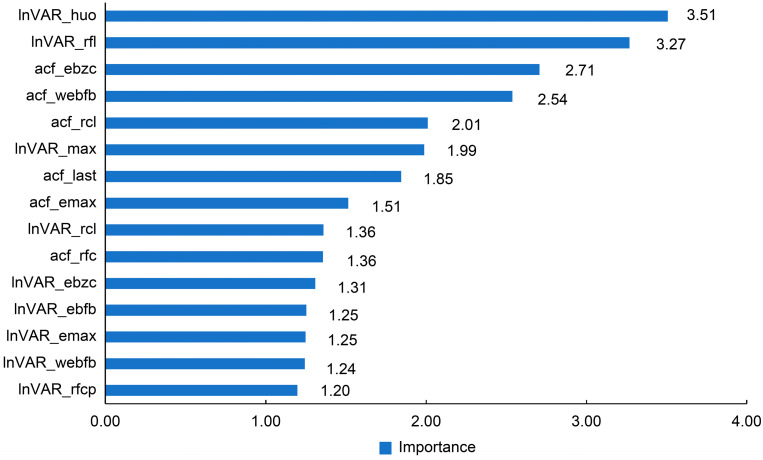
Importance of the features in partial least squares (PLS) models derived from SHAP analysis. lnVAR, log-transformed variance of deviations by quantile regression; acf, lag-1 autocorrelation fitting values; lnVAR_huo, lnVAR of daily activity; lnVAR_rfl, lnVAR of daily rumination time; lnVAR_max, lnVAR of peak value of electricity conductivity; lnVAR_rcl, lnVAR of daily milk yield; lnVAR_ebzc, lnVAR of standard deviation change of conductivity; lnVAR_ebfb, lnVAR of daily percentage change in the electrical conductivity of milk; lnVAR_emax, lnVAR of standard deviation of maximum conductivity change in last three shifts; lnVAR_webfb, lnVAR of the sum of absolute values of the weighted percentage change in the electrical conductivity of milk; lnVAR_rfcp, lnVAR of rumination deviation per 2 h; acf_ebzc, acf of standard deviation change of conductivity; acf_webfb, acf of the sum of absolute values of the weighted percentage change in the electrical conductivity of milk; acf_rcl, acf of daily milk yield; acf_last, acf of standard deviation of maximum conductivity change in last three shifts; acf_emax, acf of peak value of electricity conductivity; acf_rfc, acf of daily rumination time.

For the PLS model on the test data, the importance ranking of variables is shown in Figure 3, with the top five values being 3.51 (lnVAR_huo), 3.27 (lnVAR_rfl), 2.71 (acf_ebzc), 2.54 (acf_webfb), and 2.01 (acf_rcl).

**Figure 4:**
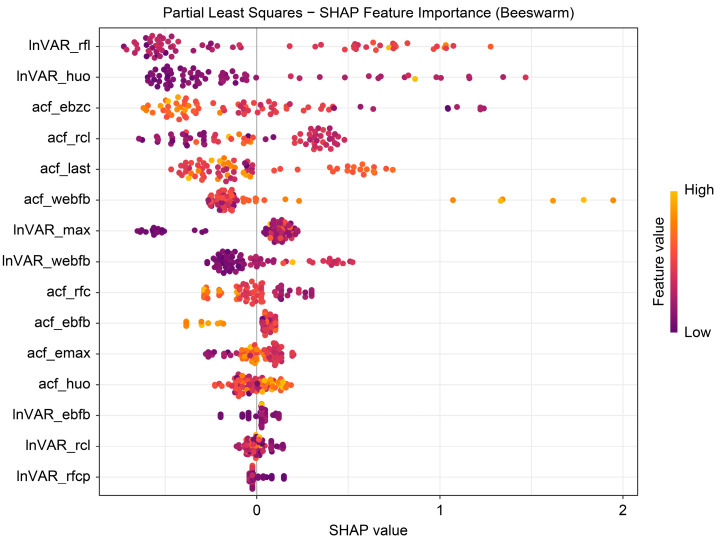
Beeswarm plot from SHAP analysis of the optimal model trained on 14-day data before mastitis onset, processed with third-order polynomial quantile regression at the 0.75 quantile. InVAR_rfI, lnVAR of daily rumination time; InVAR_huo, lnVAR of daily activity; acf_ebzc, acf of standard deviation change of conductivity; acf_rcl, acf of daily milk yield; acf_last, acf of standard deviation of maximum conductivity change in last three shifts; acf_webfb, acf of the sum of absolute values of the weighted percentage change in the electrical conductivity of milk; InVAR_max, lnVAR of peak value of electricity conductivity; InVAR_webfb, lnVAR of the sum of absolute values of the weighted percentage change in the electrical conductivity of milk; acf_rfc, acf of daily rumination time; acf_ebfb, acf of daily percentage change in the electrical conductivity of milk; acf_emax, acf of peak value of electricity conductivity; acf_huo, acf of daily activity; InVAR_ebfb, InVAR of daily percentage change in the electrical conductivity of milk; InVAR_rcl, InVAR of daily milk yield; InVAR_rfcp, lnVAR of daily rumination deviation per 2 h.

**Figure 5:**
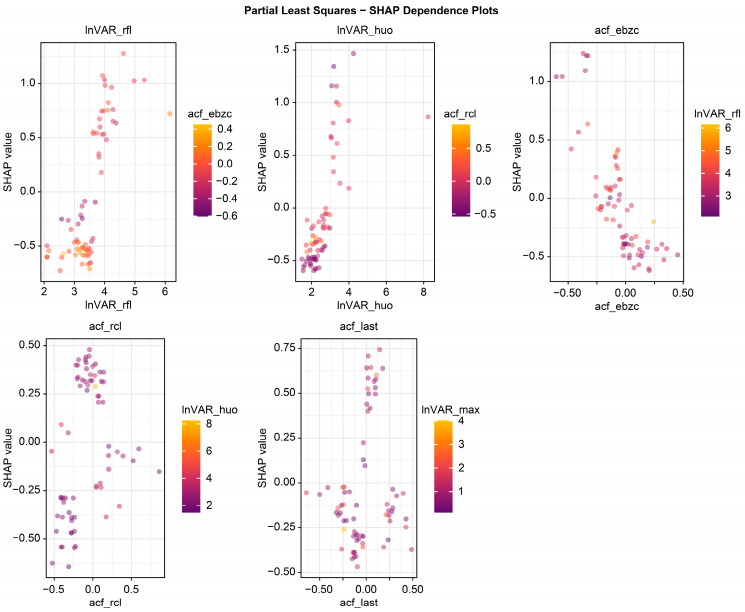
Dependence plot from SHAP analysis of the optimal model trained on 14-day data before mastitis onset, processed with third-order polynomial quantile regression at the 0.75 quantile. InVAR_rfl, InVAR of daily rumination time; InVAR_huo, lnVAR of daily activity; acf_ebzc, acf of standard deviation change of conductivity; acf_rcl, acf of daily milk yield; acf_last, acf of standard deviation of maximum conductivity change in the last three shifts.

**Figure 6:**
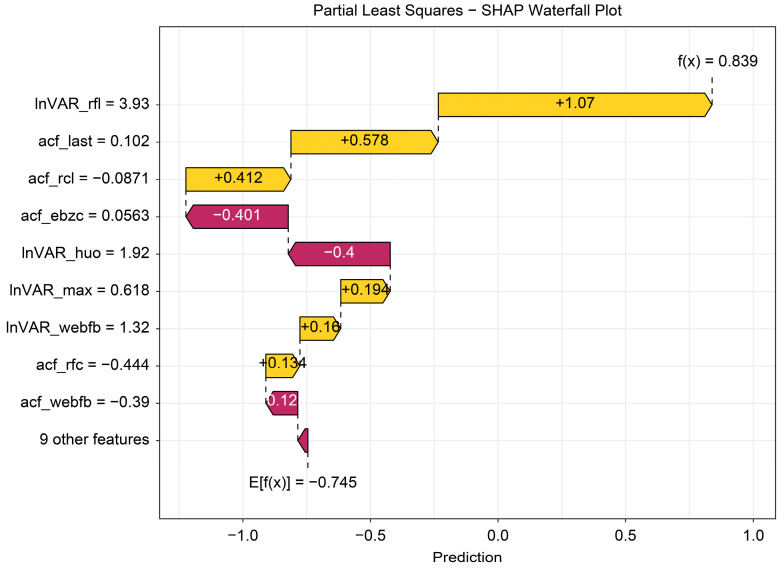
Waterfall plot from SHAP analysis of the optimal model trained on 14-day data before mastitis onset, processed with third-order polynomial quantile regression at the 0.75 quantile. The waterfall plot displays SHAP values for individual samples by ranking feature contributions, intuitively visualizing how each variable influenced the prediction. The value “E[f(x)] = −0.745” at the base of the graph represents the mean of all sample predictions (the baseline without sample-specific features), whereas the value “f(x) = 0.839” at the top reflects the final prediction after accounting for feature contributions. Feature names and values listed on the left correspond to their SHAP impacts, with yellow arrows indicating increases in prediction and red arrows indicating decreases. InVAR_rfI, InVAR of daily rumination time; acf_last, acf of standard deviation of maximum conductivity change in last three shifts; acf_rcl, acf of daily milk yield; acf_ebzc, acf of standard deviation change of conductivity; InVAR_huo, lnVAR of daily activity; InVAR_max, InVAR of peak value of electricity conductivity; InVAR_webfb, InVAR of the sum of absolute values of the weighted percentage change in the electrical conductivity of milk; acf_rfc, acf of daily rumination time; acf_webfb, acf of the sum of absolute values of the weighted percentage change in the electrical conductivity of milk.

**Figure 7:**
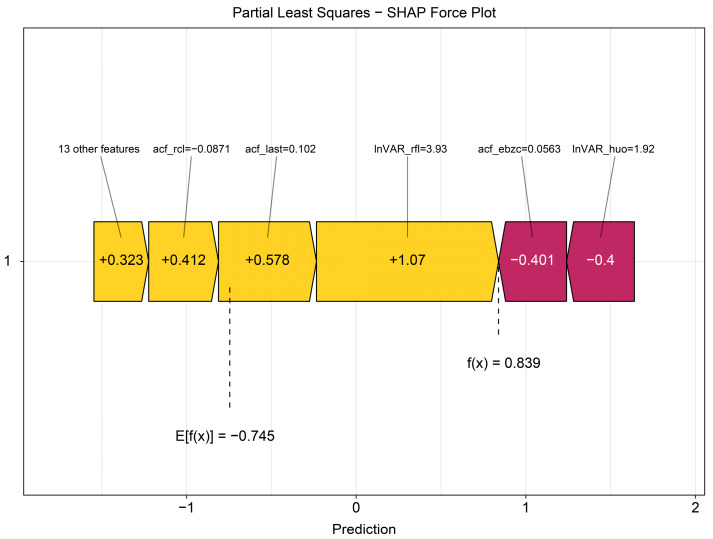
Force plot from SHAP analysis of the optimal model trained on 14-day data before mastitis onset, processed with third-order polynomial quantile regression at the 0.75 quantile. InVAR_rfl, InVAR of daily rumination time; InVAR_huo, lnVAR of daily activity; acf_ebzc, acf of standard deviation change of conductivity; acf_rcl, acf of daily milk yield; acf_last, acf of standard deviation of maximum conductivity change in last three shifts.

The beeswarm plot (Figure 4) highlights InVAR_rfl and acf_last as positive contributors and acf_ebzc and lnVAR_huo as negative contributors. The dependence plot (Figure 5) demonstrates nonlinear associations between SHAP values and the top five variables (lnVAR_rfl, lnVAR_huo, acf_ebzc, acf_rcl, and acf_last) for mastitis prediction. The waterfall plot (Figure 6) illustrates feature effects and interaction directions: yellow (right) arrows represent positive prediction impact, and purple (left) arrows represent negative impact, with arrow length reflecting effect strength. Six of the nine features increased prediction probability (higher feature value, higher prediction), with the top three SHAP values being 1.07, 0.578, and 0.412, for lnVAR_rfl, acf_last, and acf_rcl, respectively. In contrast, acf_ebzc (−0.401) and lnVAR_huo (−0.400) consistently negatively influenced prediction. The contribution of each key feature is depicted in Figure 7.

SHAP analysis of the optimal models for 7 (second-order, 0.5 quantile), 21 (second-order, 0.5 quantile), and 28 days (second-order, 0.75 quantile) before mastitis onset are presented in Supplementary Figures S4–S6. Two of the variables, acf_rcl and lnVAR_huo, appeared in all four periods, whereas acf_rcl and lnVAR_ebzc contributed positively across three periods. Notably, lnVAR_huo showed a consistent negative contribution across all time windows.

## 4. Discussion

Timely and accurate prediction of mastitis is essential to facilitate early treatment, safeguard animal welfare, and mitigate the short- and long-term impacts of the disease on productivity and the environment. However, analyzing real-time data from farm sensors, monitoring systems, and management software presents challenges due to their high-dimensional structure and typically limited number of animals or samples, leading to data imbalance and an increased risk of overfitting in many ML models. Consequently, greater emphasis must be placed on processing preliminary data, filtering statistically significant variables, and optimizing model hyperparameters.

Quantile regression estimates parameters by minimizing absolute, rather than squared, errors, making it less sensitive to outliers and thus yielding more robust results. By not assuming any specific data distribution, QR also relaxes the requirements on data patterns, making it particularly suitable for non-normally distributed datasets. For large datasets requiring fast and accessible analysis, QR may be an optimal choice.

Kok et al. [8] utilized QR to derive quantile residuals of daily milk yield, which were used to assess two traits: lnVAR and lag-1 autocorrelation, i.e., the correlation between quantile residuals on subsequent days during the evaluation period. Similarly, the present study employed QR to obtain quantile residuals for daily activity, daily rumination time, and daily electrical conductivity, in addition to daily milk yield, to generate more reliable input for determining whether subtle variations in these parameters reflect the mammary gland’s health status, rather than relying solely on raw daily real-time data. Consistent with the findings of Kok et al. [8], we found that in the 28 days preceding mastitis onset, the autocorrelation of daily milk yield was positively correlated with lnVAR in both mastitis-affected and healthy cows, with correlation coefficients of 0.390 (*p* = 0.023) and 0.328 (*p* = 0.008), respectively. Additionally, lnVAR_rcl was significantly higher in mastitic cows than in healthy cows (0.28 ± 0.11, *p* = 0.01). For shorter time windows (7, 14, and 21 days), the autocorrelation of daily milk yield was not correlated with lnVAR in either group; however, lnVAR_rcl remained elevated in mastitis cows (7 days: 0.55 ± 0.19, *p* < 0.01; 14 days: 0.70 ± 0.17, *p* < 0.01; 21 days: 0.29 ± 0.11, *p* = 0.014). The development of clinical mastitis was associated with increased lnVAR_rcl (log-transformed variance of deviations in daily milk yield), consistent with the results of Kok et al. [8]. This relationship is further supported by the SHAP waterfall plots (Supplementary Figures S4D and S5D) and reduced acf_rcl (autocorrelation of deviations in milk yield; Supplementary Figure S6D).

In this study, raw data obtained from automated monitoring and intelligent farm management systems were first split into a training set (70% of all data) and a test set (30% of all data) to ensure the analysis at the cow level to avoid the potential information leakage, and then processed using QR, followed by variable significance using binary logistic regression. Eleven widely used ML algorithms were then employed to construct the mastitis prediction models. SHAP analysis was subsequently applied to generate both global and local interpretable visualizations, thereby enhancing model transparency and elucidating the direction and magnitude of influence exerted by each variable on the predictive outcome. SHAP values originate from Shapley values in cooperative game theory, which satisfy four fundamental axioms: efficiency, symmetry, dummy and additivity, constituting the theoretical foundation for applying this method to the machine learning models across the four time windows considered in the current study. Machine learning offers the advantage of autonomously uncovering nonlinear relationships and interactions in multi-dimensional datasets, surpassing the limitations of traditional single-factor approaches. Rowe et al. [16] compared a conventional rule-based algorithm for selective dry cow therapy with six ML models for detecting intramammary infections and found that the RF model outperformed the others based on the evaluation metrics used. Our study covered all six models evaluated by Rowe et al. [16]. Using milk parameters and animal-level data, Satoła and Satoła [17] compared several ML models, including bagging, boosting, stacking, and super-learner ensembles, with single-model approaches to predict subclinical mastitis. Their findings showed that the super learner exhibited superior predictive accuracy. Thompson et al. [18] utilized the XGBoost algorithm to probabilistically forecast the risk of elevated somatic cell count (SCC) in individual cows post-calving, demonstrating that reliable classification of elevated SCC risk within 30 days of calving is achievable. Luo et al. [19] evaluated four ML algorithms—DT, RF, backpropagation neural networks, and SVM—and reported that the DT algorithm achieved the highest accuracy. Similarly, Yesil and Goncu [20] developed artificial neural networks (ANNs) to classify mastitis severity into four categories using five milk-derived features: SCC, EC, pH, density, and pre-milking temperature. Nagy et al. [21] demonstrated that training ANNs on data from conventional milking systems improved the diagnosis of subclinical mastitis. Their best-performing ANN achieved a sensitivity of 0.54 and specificity of 0.77. In contrast, the optimal model in our study, which used second- and third-order polynomial features at the 0.5 and 0.75 quantiles, achieved a specificity exceeding 0.84 across all four time periods, though sensitivity remained at the 0.500. Guo et al. [22] assessed six ML models using time-series features derived from production indicators for each cow across two consecutive months to predict health status in the third month. Among the models tested, XGBoost again delivered the best performance. SHAP value analysis validated the predictive importance of these temporal features.

Few studies have applied SHAP analysis to predict and diagnose dairy cow diseases [13,23,24], classify dairy cow behavior [25,26], and predict the impact of heat stress on behavior [27]. SHAP explains ML predictions through SHAP values, clarifying which features influence a specific prediction, their direction (positive or negative), and the magnitude of their impact. Although many studies have integrated multiple ML algorithms to construct prediction models, reports incorporating SHAP interpretability remain scarce. Global interpretability is typically assessed using feature importance and beeswarm plots. In the current study, the feature lnVAR_huo, consistently selected across all four optimal models, exerted the greatest influence on predictions. The beeswarm plot showed that lnVAR_huo had the widest SHAP value distribution, indicating a significant impact and predominantly strong negative effect, followed by acf_rcl. The broad SHAP value distribution of lnVAR_huo highlights its critical role in bovine mastitis prediction. Minor activity variations may reflect dairy cow health status—udders experiencing discomfort correlate with increased lying time, reduced lying frequency, and shorter activity duration. Subtle deviations in milk yield (acf_rcl: autocorrelation value of daily milk yield, numerical metrics quantifying similarity between a time series and its lagged versions) also indicate udder health and potential disease risk. Additionally, the lnVAR of electrical conductivity variability serves as a risk-enhancing feature, consistent with elevated conductivity signaling bovine mastitis risk. High feature values (depicted in orange) generally corresponded with positive SHAP values, suggesting a positive contribution to the predicted outcome, as observed for both acf_rcl and lnVAR_webfb. Local interpretability was demonstrated using waterfall and force plots, which are particularly useful for explaining model decisions to clinical decision-makers. Similarly, force plots represent positive and negative contributions using arrows—yellow (rightward) for positive and purple (leftward) for negative. The length of the arrow reflects the magnitude of each feature’s effect [12,13,14,23,26].

This study has some limitations. Although k-fold cross-validation was employed to assess model stability, sensitivity remained relatively low—leading to missed detection of half the mastitic cows, likely due to class imbalance, limited sample size, or the noisy nature of real-world data. False negative results mean infected cows do not receive timely treatment, prolonging their pain and discomfort. Mastitis itself causes symptoms such as breast swelling, pain, and fever in the udder. Undiagnosed cases also lead to continuous declines in milk production and quality. Untreated cases may progress to chronic infections or systemic diseases, further compromising animal welfare. Additionally, they may result in reduced reproductive performance [5] (such as lower conception rates, delayed ovulation, or anovulation). Long-term untreated infections can increase culling rates and shorten the productive lifespan of dairy cows, thereby causing a rise in feeding costs and management expenses [17].

With smaller datasets, internal validation performance assessments may be overly optimistic, primarily due to evaluation noise caused by insufficient sample size, leading to a decline in generalization ability. Several models show wide confidence intervals for AUC values, which indicates model instability. In the ongoing study on analyzing other disorders, we will consider implementing several measures to enhance sensitivity and thus improve the model’s stability and practical utility. These measures include: augmenting the quantity or quality of training data to boost model performance; selecting more discriminative features or engineering new ones to facilitate better signal learning for machine learning models; optimizing hyperparameters [28] (e.g., via grid search, random search, or Bayesian optimization to identify the optimal parameter combination); and enhancing model performance through ensemble learning, etc. Lowering the threshold [29] may be attempted to improve the model’s ability to identify the positive class, thereby increasing recall (true positive rate). The challenge of the ongoing work is to balance these two model metrics to enhance practicality. In addition, external validation has not yet been performed owing to time constraints and sample collection challenges (few farms raising cows milked four times per day, applying parallel milking parlors). Such validation will enhance the credibility and reliability of the prediction methods and models. In future research, aside from increasing the sample size, regularization, dropout, and early training termination could also be tried to alleviate overfitting. Anyhow, external validation on one or more independent herds, ideally with different conditions (such as breeds, milking schedules, management, or sensors), is necessary before the model is implemented in practice. For the dataset analyzed in the current study, the 14-day window is the most promising. Future research will increase the sample size and focus on hyperparameter tuning of the machine learning models to improve performance over longer prediction windows.

## 5. Conclusions

To the best of our knowledge, this is the first study to apply QR analysis to preprocess raw auto-monitoring data by combining different quantiles and polynomial orders, in addition to using lnVAR of daily milk yield to predict clinical mastitis. After identifying the optimal ML models, SHAP visualizations were used to quantify the predictive contribution of each variable, helping overcome the “black box” nature of ML models.

The lnVAR and acf features were calculated using QR across four distinct time windows, applying second- or third-order polynomials at the 0.5 and 0.75 quantiles of the raw data. This approach enabled robust modeling of variable distributions under varying operational conditions. Variables significantly associated with mastitis were identified using binary logistic regression. Eleven widely used ML algorithms were then employed to train mastitis prediction models. Among them, the PLS model based on 14-day data processed with third-order polynomial QR at the 0.75 quantile demonstrated superior performance across six evaluation metrics. Finally, SHAP value analysis highlighted acf_rcl, lnVAR_ebzc, and lnVAR_huo as the most indicative features. Overall, the findings offer an interpretable framework for early bovine mastitis detection and provide a theoretical foundation for intelligent monitoring and developing mastitis prediction software. To strengthen the practical application of the study, future work will focus on enhancing model performance by expanding the sample size, incorporating non-invasive physiological and biochemical indicators, setting hyperparameters and tuning thresholds of ML models on real-time data, and so on.

## Figures and Tables

**Table 1:** Number of records for the data of cows analyzed in the experiment.

Sensor/ Milking System	Trait	Variable	Measurements Interval	Unit	No.
Automatic monitoring system	Activity	Activity	Min/2 h	Min/day	5936
	Rumination	Daily Rumination time	Min/2 h	Min/day	5936
		Rumination deviation per 2 h	Min/2 h	Min/day	71,272
		Sum of the absolute values of the weighted rumination variation	No/2 h	Min/day	71,272
Parallel milking system	Milk yield	Milk yield	Four times/day	Kg/day	5936
	Parity	Parity	No/year	Non	222
	Milk lactation	DIM	No/day	Day	222
	Milk electrical conductivity	Peak electrical conductivity of milk	No/milking shift	Non	23,744
		Daily percentage change in the electrical conductivity of milk	mS/cm/milking shift	mS/cm	23,744
		Standard deviation change in conductivity	mS/cm/milking shift	mS/cm	23,744
		Standard deviation of the maximum conductivity change in the last three shifts	mS/cm/milking shift	mS/cm	23,744
Total					255,772

Min, minute; h, hour; No, number; mS, millisiemens; cm, centimeter; Kg, kilogram; non, none.

**Table 2:** Comparison of the top nine features from univariate and multivariate analyses of quantile regression on training data 14 days before mastitis onset (third-order polynomial quantile regression at the 0.75 quantile).

Feature	Mastitis (No = 48, 30.6%)	Healthy (No = 108, 69.4%)	*p*-Value Univariate	*p*-Value Multivariate
lnVAR_rfl (mean (SD))	3.88 (0.79)	3.31 (0.56)	0.001	0.02
acf_last (mean (SD))	−0.03 (0.23)	−0.02 (0.23)	0.023	0.049
acf_rcl (mean (SD))	−0.07 (0.20)	−0.09 (0.31)	0.015	0.043
acf_ebzc (mean (SD))	−0.07 (0.19)	−0.07 (0.23)	0.042	0.049
lnVAR_huo (mean (SD))	2.98 (1.30)	2.35 (0.55)	<0.001	<0.001
lnVAR_max (mean (SD))	1.70 (0.64)	1.83 (0.80)	<0.001	<0.001
lnVAR_webfb (mean (SD))	1.19 (0.84)	0.89 (0.47)	<0.001	<0.001
acf_rfc (mean (SD))	−0.10 (0.22)	−0.08 (0.22)	<0.001	<0.001
acf_webfb (mean (SD))	0.01 (0.29)	−0.06 (0.19)	<0.001	<0.001

No, number. lnVAR, log-transformed variance of deviations by quantile regression; acf, lag-1 autocorrelation fitting values; lnVAR_rfl, lnVAR of daily rumination time; acf_last, acf of standard deviation of maximum conductivity change in last three shifts; acf_rcl, acf of daily milk yield; acf_ebzc, acf of standard deviation change of conductivity; lnVAR_huo, lnVAR of daily activity; lnVAR_max, lnVAR of peak value of electricity conductivity; lnVAR_webfb, lnVAR of the sum of absolute values of the weighted percentage change in the electrical conductivity of milk; acf_rfc, acf of daily rumination time; acf_webfb, acf of the sum of absolute values of the weighted percentage change in the electrical conductivity of milk; SD, standard deviation.

**Table 3:** Performance metrics of optimal ML models across four-time windows using second/third order quantile regression with a median (0.50)/0.75 quantile.

Days	Order	Quantile	Model	AUC	Accuracy	Sensitivity	Specificity	Precision	F1_Score
7	2	0.50	**LDA**	0.758	0.724	0.500	0.842	0.625	0.556
				([0.572, 0.943])	([0.642, 0.845])	([0.300, 0.700])	([0.742, 0.938])	([0.386, 0.815])	([0.350, 0.740])
7	2	0.75	SVM	0.647	0.724	0.300	0.947	0.750	0.429
				([0.405, 0.890])	([0.643, 0.847])	([0.113, 0.512])	([0.852, 0.987])	([0.312, 0.932])	([0.338, 0.579])
7	3	0.50	PLS	0.663	0.724	0.400	0.895	0.667	0.500
				([0.433, 0.894])	([0.643, 0.847])	([0.177, 0.613])	([0.785, 0.966])	([0.377, 0.885])	([0.383, 0.633])
7	3	0.75	KNN	0.674	0.655	0.300	0.842	0.500	0.375
				([0.457, 0.890])	([0.554, 0.784])	([0.113, 0.512])	([0.712, 0.924])	([0.215, 0.757])	([0.287, 0.514])
14	2	0.50	AdaBoost	0.661	0.690	0.500	0.789	0.556	0.526
				([0.450, 0.871])	([0.607, 0.823])	([0.287, 0.713])	([0.691, 0.911])	([0.313, 0.774])	([0.423, 0.624])
14	2	0.75	KNN	0.821	0.621	0.500	0.684	0.455	0.476
				([0.670, 0.973])	([0.502, 0.737])	([0.287, 0.713])	([0.528, 0.798])	([0.237, 0.587])	([0.358, 0.547])
14	3	0.50	RF	0.745	0.690	0.300	0.895	0.600	0.400
				([0.562, 0.927])	([0.593, 0.813])	([0.137, 0.527])	([0.764, 0.957])	([0.232, 0.812])	([0.285, 0.503])
14	3	0.75	**PLS**	0.789	0.793	0.500	0.947	0.833	0.625
				([0.611, 0.968])	([0.713, 0.895])	([0.287, 0.713])	([0.858, 0.989])	([0.526, 0.974])	([0.523, 0.727])
21	2	0.50	PLS	0.789	0.724	0.500	0.842	0.625	0.556
				([0.624, 0.955])	([0.625, 0.838])	([0.287, 0.713])	([0.723, 0.927])	([0.345, 0.803])	([0.442, 0.643])
21	2	0.75	**AdaBoost**	0.637	0.759	0.400	0.947	0.800	0.533
				([0.405, 0.869])	([0.675, 0.873])	([0.195, 0.605])	([0.858, 0.989])	([0.441, 0.975])	([0.372, 0.672])
21	3	0.50	LDA	0.763	0.690	0.300	0.895	0.600	0.400
				([0.540, 0.986])	([0.583, 0.818])	([0.103, 0.497])	([0.762, 0.955])	([0.288, 0.787])	([0.278, 0.524])
21	3	0.75	RF	0.637	0.655	0.400	0.895	0.500	0.286
				([0.422, 0.852])	([0.637, 0.837])	([0.195, 0.605])	([0.762, 0.955])	([0.332, 0.835])	([0.342, 0.624])
28	2	0.50	PLS	0.637	0.724	0.300	0.947	0.750	0.429
				([0.409, 0.864])	([0.658, 0.848])	([0.103, 0.497])	([0.868, 0.991])	([0.374, 0.955])	([0.278, 0.524])
28	2	0.75	**GLM**	0.668	0.724	0.500	0.842	0.625	0.556
				([0.446, 0.891])	([0.647, 0.838])	([0.296, 0.704])	([0.725, 0.926])	([0.358, 0.819])	([0.447, 0.648])
28	3	0.50	PLS	0.626	0.690	0.200	0.947	0.667	0.308
				([0.409, 0.844])	([0.613, 0.820])	([0.032, 0.368])	([0.868, 0.991])	([0.253, 0.923])	([0.151, 0.373])
28	3	0.75	AdaBoost	0.624	0.655	0.200	0.895	0.500	0.286
				([0.417, 0.830])	([0.564, 0.782])	([0.564, 0.782])	([0.779, 0.954])	([0.133, 0.755])	([0.121, 0.312])

LDA: linear discriminant analysis; SVM: support vector machine; PLS: partial least squares; KNN: k-nearest neighbors; AdaBoost: AdaBoost; RF: random forest; GLM: logistic regression analysis; AUC: area under the ROC curve.

## Data Availability

Data and procedure will be made available upon request to the corresponding author.

## Supplementary Materials

The following supporting information can be downloaded at https://www.mdpi.com/article/10.3390/ani16020204/s1, Figure S1: Receiver operating characteristic (ROC) curves of 11 machine learning (ML) models trained on 7-day data before mastitis onset, processed with second-order polynomial quantile regression at the 0.5 quantile; Figure S2: Receiver operating characteristic (ROC) curves of 11 machine learning (ML) models trained on 21-day data before mastitis onset, processed with second-order polynomial quantile regression at the 0.5 quantile; Figure S3: Receiver operating characteristic (ROC) curves of 11 machine learning (ML) models trained on 28-day data before mastitis onset, processed with second-order polynomial quantile regression at the 0.75 quantile; Figure S4: SHAP analysis for the linear discriminant analysis model on the data of 7-day window with second-order polynomial quantile regression at the 0.5 quantile; Figure S5: SHAP analysis for the linear discriminant analysis model on the data of 21-day window with second-order polynomial quantile regression at the 0.5 quantile; Figure S6: SHAP analysis for the logistic regression model on the data of 28-day window with second-order polynomial quantile regression at the 0.75 quantile; Table S1: Comparisons of key features from univariate and multivariate analyses for the 7-, 21-, and 28-day windows (second/third-order polynomial quantile regression at the 0.5/0.75 quantiles) between mastitic and healthy cows. Table S2. Abbreviation and full names for features in the analysis of the PLS model.
