# Supplementary material for: Machine Learning and SHapley Additive exPlanation-Based Interpretation for Predicting Mastitis in Dairy Cows
Source: Animals (Basel). 2026 Jan 9;16(2):204. doi: 10.3390/ani16020204 (PMC12837133; doi:10.3390/ani16020204)
Supplement: Supplementary file 1 [file animals-16-00204-s001.zip › animals-4030796-supplementary.pdf]

Supplementary Material (available at https://osf.io/ub3wa)

Supplementary Figures

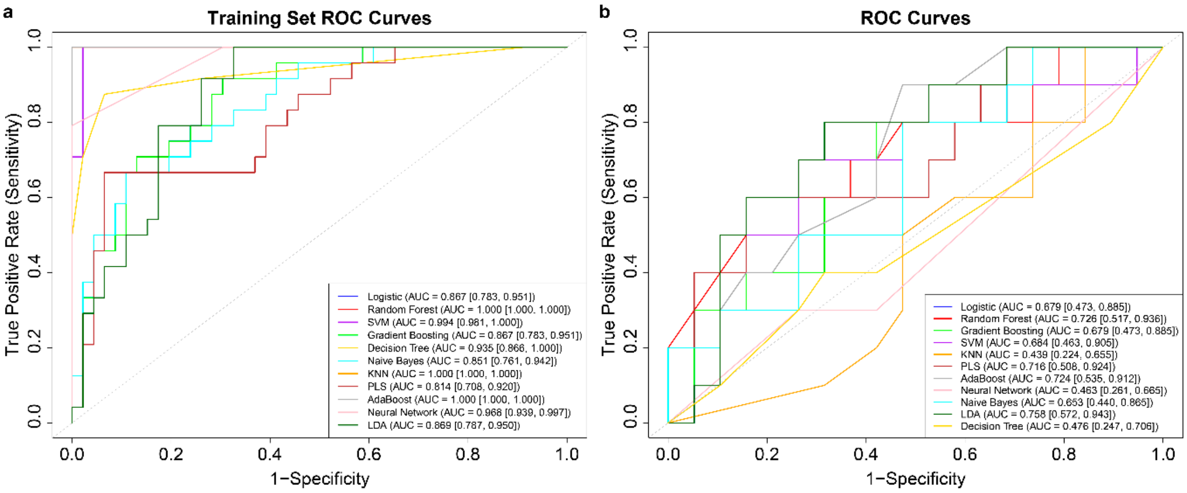

Figure S1. Receiver operating characteristic (ROC) curves of 11 machine learning (ML) models trained on 7-day data before mastitis onset, processed using second-order polynomial quantile regression at the 0.5 quantile**: (A**) ROC curves of the 11 ML algorithms on the training data, with 95% confidence intervals of ROC-AUC obtained via cross-validation shown in the bottom right corner; (**B**) ROC curves of the 11 ML algorithms on the corresponding test data, with 95% confidence intervals of ROC-AUC obtained via cross-validation shown in the bottom right corner. Abbreviations: Logistic, logistic regression analysis; RF, random forest; SVM, support vector machine; GB, gradient boost; DT, decision tree; NB, naive bayes; KNN, k-nearest neighbors; PLS, partial least squares; Adaboost, Adaboost; NN, neural network; LDA, linear discriminant analysis; ROC, receiver operating characteristic curve; AUC, area under ROC.

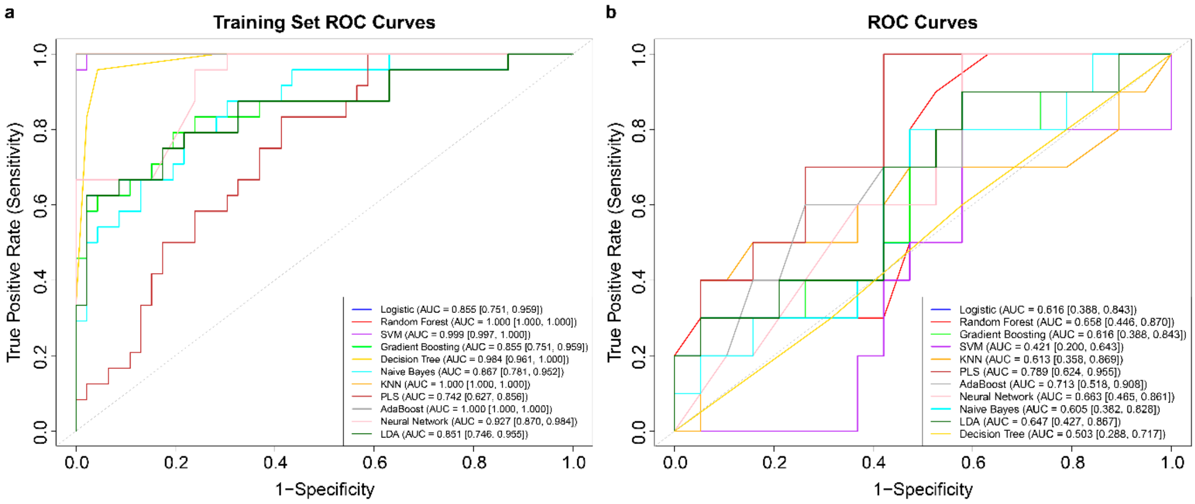

Figure S2. Receiver operating characteristic (ROC) curves of 11 machine learning (ML) models trained on 21-day data before mastitis onset, processed using second-order polynomial quantile regression at the 0.5 quantile**:** (**A**) ROC curves of the 11 ML algorithms on the training data, with 95% confidence intervals of ROC-AUC obtained via cross-validation shown in the bottom right corner**;** (**B**) ROC curves of the 11 ML algorithms on the corresponding test data, with 95% confidence intervals of ROC-AUC obtained via cross-validation shown in the bottom right corner. Abbreviations: Logistic, logistic regression analysis; RF, random forest; SVM, support vector machine; GB, gradient boost; DT, decision tree; NB, naive bayes; KNN, k-nearest neighbors; PLS, partial least squares; Adaboost, Adaboost; NN, neural network; LDA, linear discriminant analysis; ROC, receiver operating characteristic curve; AUC, area under ROC.

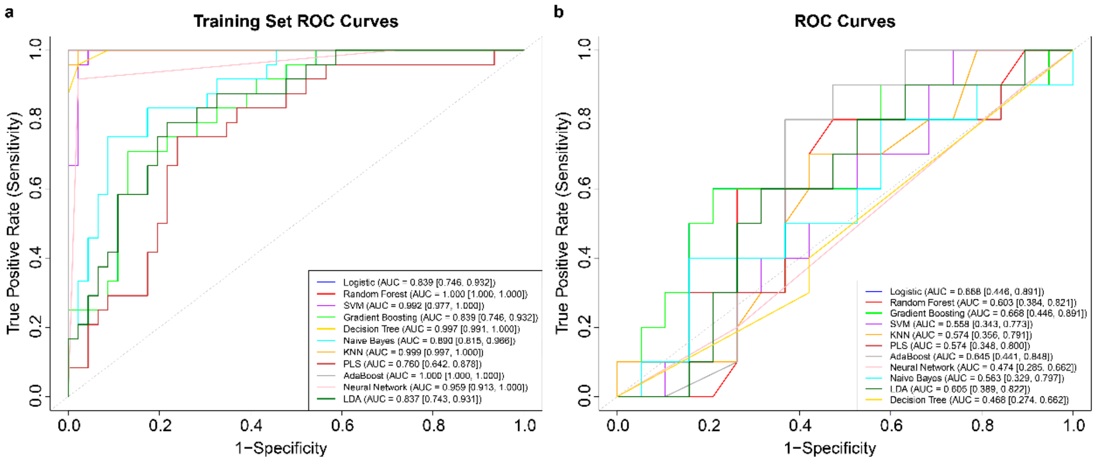

Figure S3. Receiver operating characteristic (ROC) curves of 11 machine learning (ML) models trained on 28-day data before mastitis onset, processed using second-order polynomial quantile regression at the 0.75 quantile**:** (**A**) ROC curves of the 11 ML algorithms on the training data, with 95% confidence intervals of ROC-AUC obtained via cross-validation shown in the bottom right corner: (**B**) ROC curves of the 11 ML algorithms on the corresponding test data, with 95% confidence intervals of ROC-AUC obtained via cross-validation shown in the bottom right corner. Abbreviations: Logistic, logistic regression analysis; RF, random forest; SVM support vector machine; GB, gradient boost; DT, decision tree; NB, naive bayes; KNN, k-nearest neighbors; PLS, partial least squares; Adaboost, Adaboost; NN, neural network; LDA, linear discriminant analysis; ROC, receiver operating characteristic curve; AUC, area under ROC.

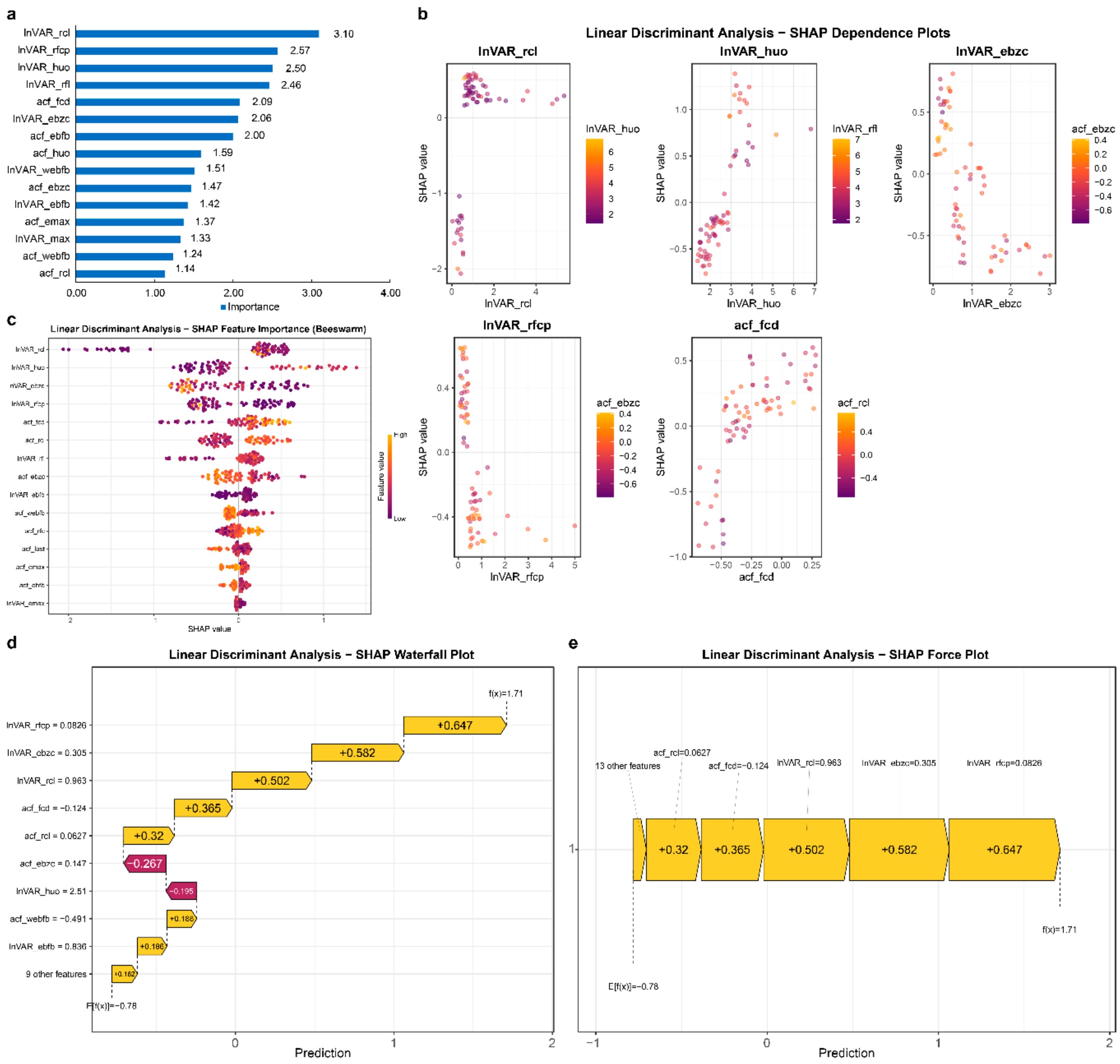

Figure S4. SHAP analysis for the linear discriminant analysis model trained on 7-day data processed using second-order polynomial quantile regression at the 0.5 quantile: (**A**) Feature importance from SHAP analysis for the optimal model (7-day window, second-order polynomial quantile regression at the 0.5 quantile). LnVAR_huo, log-transformed variance of deviations (lnVAR) by quantile regression for variable of daily activity; lnVAR_rfl, lnVAR of daily rumination time; lnVAR_max, lnVAR of peak value of electricity conductivity; lnVAR_rcl, lnVAR of daily milk yield; lnVAR_ebzc, lnVAR of standard deviation change of conductivity; lnVAR_ebfb, lnVAR of conductivity variation percentage; lnVAR_emax, lnVAR of standard deviation of maximum conductivity change in last three shifts; lnVAR_webfb, lnVAR of the sum of absolute values of the weighted rumination variation; lnVAR_rfcp, lnVAR of daily rumination deviation per 2 h; acf_ebzc, lag-1 auto-correlation values (acf) of standard deviation change of conductivity; acf_webfb, acf of the sum of absolute values of the weighted rumination variation; acf_rcl, acf of daily milk yield; acf_last, acf of standard deviation of maximum conductivity change in last three shifts; acf_emax, acf of peak value of electricity conductivity; acf_rfc, acf of daily rumination time. (**B**) SHAP dependence plots for the optimal model**;** (**C**) SHAP beeswarm plot; (**D**) SHAP waterfall plot; (**E**) SHAP force plot.

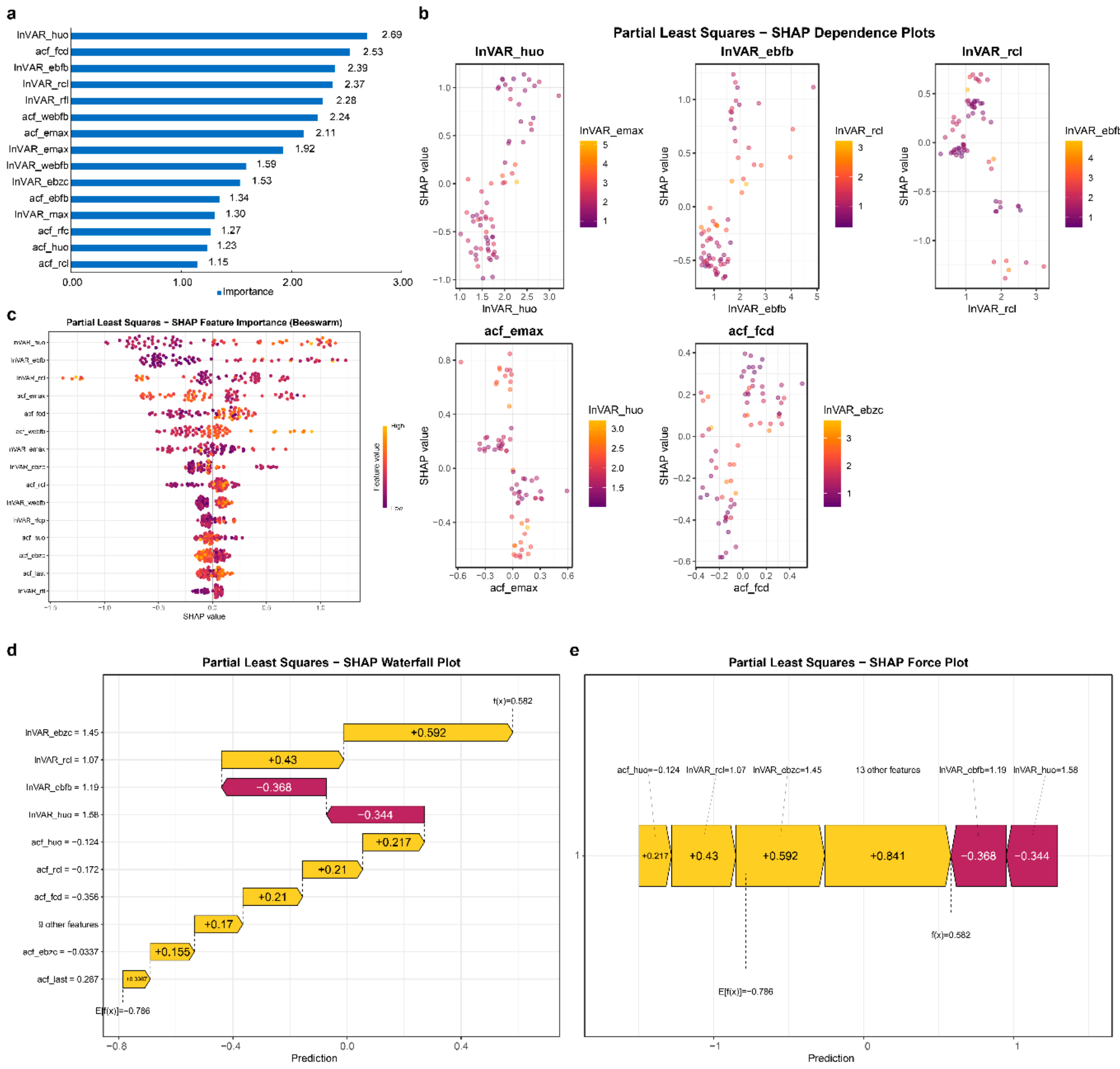

Figure S5. SHAP analysis for the linear discriminant analysis model trained on 21-day data processed using second-order polynomial quantile regression at the 0.5 quantile. (**A**) Feature importance from SHAP analysis for the optimal model (21-day window, second-order polynomial quantile regression at the 0.5 quantile). LnVAR_huo, log-transformed variance of deviations (lnVAR) by quantile regression for variable of daily activity; lnVAR_rfl, lnVAR of daily rumination time; lnVAR_max, lnVAR of peak value of electricity conductivity; lnVAR_rcl, lnVAR of daily milk yield; lnVAR_ebzc, lnVAR of standard deviation change of conductivity; lnVAR_ebfb, lnVAR of conductivity variation percentage; lnVAR_emax, lnVAR of standard deviation of maximum conductivity change in last three shifts; lnVAR_webfb, lnVAR of the sum of absolute values of the weighted rumination variation; lnVAR_rfcp, lnVAR of daily rumination deviation per 2 h; acf_ebzc, lag-1 auto-correlation values (acf) of standard deviation change of conductivity; acf_webfb, acf of the sum of absolute values of the weighted rumination variation; acf_rcl, acf of daily milk yield; acf_last, acf of standard deviation of maximum conductivity change in last three shifts; acf_emax, acf of peak value of electricity conductivity; acf_rfc, acf of daily rumination time. (**B**) SHAP dependence plots for the optimal model; (**C**) SHAP beeswarm plot; (**D**) SHAP waterfall plot; (**E**) SHAP force plot.

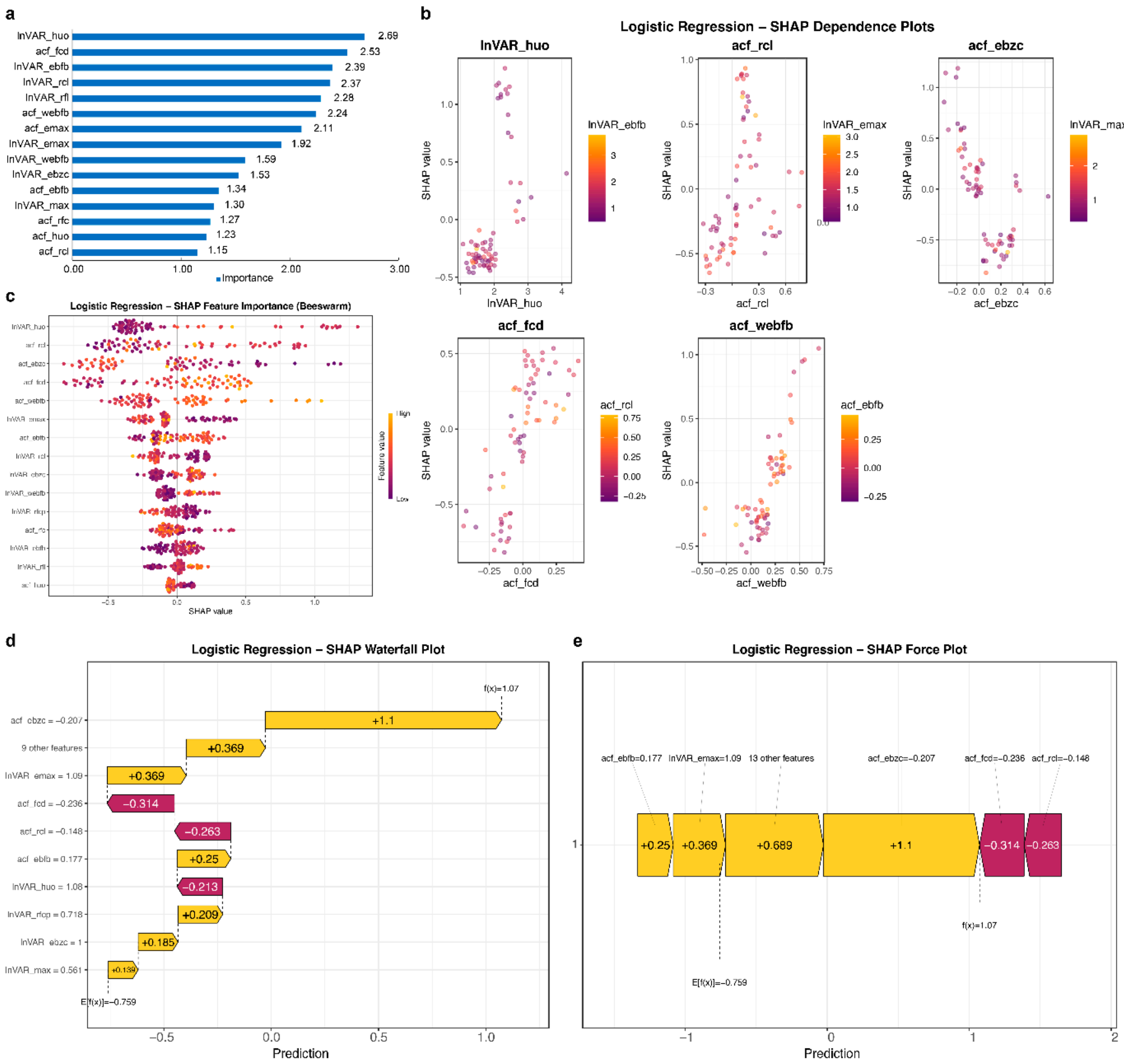

Figure S6. SHAP analysis for the logistic regression model trained on 28-day data processed using second-order polynomial quantile regression at the 0.75 quantile**:** (**A**) Feature importance from SHAP analysis for the optimal model (28-day window, second-order polynomial quantile regression at the 0.75 quantile). LnVAR_huo, log-transformed variance of deviations (lnVAR) by quantile regression for variable of daily activity; lnVAR_rfl, lnVAR of daily rumination time; lnVAR_max, lnVAR of peak value of electricity conductivity; lnVAR_rcl, lnVAR of daily milk yield; lnVAR_ebzc, lnVAR of standard deviation change of conductivity; lnVAR_ebfb, lnVAR of conductivity variation percentage; lnVAR_emax, lnVAR of standard deviation of maximum conductivity change in last three shifts; lnVAR_webfb, lnVAR of the sum of absolute values of the weighted rumination variation; lnVAR_rfcp, lnVAR of daily rumination deviation per 2 h; acf_ebzc, lag-1 auto-correlation values (acf) of standard deviation change of conductivity; acf_webfb, acf of the sum of absolute values of the weighted rumination variation; acf_rcl, acf of daily milk yield; acf_last, acf of standard deviation of maximum conductivity change in last three shifts; acf_emax, acf of peak value of electricity conductivity; acf_rfc, acf of daily rumination time. (**B**) SHAP dependence plots for the optimal model; (**C**) SHAP beeswarm plot; (**D**) SHAP waterfall plot; (**E**) SHAP force plot.

Supplementary Table

Table S1. Comparisons of key features from univariate and multivariate analyses for the 7-, 21-, and 28-day windows (second/third-order polynomial quantile regression at 0.5/0.75 quantiles) between mastitic and healthy cows.

Characteristic	Mastitis (N = 48, 30.6%)	Healthy (N = 108, 69.4%)	P value univariate	P value multivariate
**LDA: 7d of a second-order polynomial quantile regression with a median 0.50**				
lnVAR_rfl (mean (SD))	3.88 (0.79)	3.31 (0.56)	0.001	0.02
acf_last (mean (SD))	-0.03 (0.23)	-0.02 (0.23)	0.009	0.045
acf_rcl (mean (SD))	-0.07 (0.20)	-0.09 (0.31)	0.01	0.043
acf_ebzc (mean (SD))	-0.07 (0.19)	-0.07 (0.23)	0.045	0.236
lnVAR_huo (mean (SD))	2.98 (1.30)	2.35 (0.55)	p<0.001	p<0.001
lnVAR_max (mean (SD))	1.70 (0.64)	1.83 (0.80)	p<0.001	p<0.001
lnVAR_webfb (mean (SD))	1.19 (0.84)	0.89 (0.47)	p<0.001	p<0.001
acf_rfc (mean (SD))	-0.10 (0.22)	-0.08 (0.22)	0.035	0.07
acf_webfb (mean (SD))	0.01 (0.29)	-0.06 (0.19)	p<0.001	p<0.001
**PLS: 21d of a second-order polynomial quantile regression with a median 0.50**				
acf_emax (mean (SD))	-0.03 (0.18)	0.01 (0.19)	p<0.001	p<0.001
acf_ebfb (mean (SD))	-0.02 (0.17)	0.01 (0.20)	p<0.001	p<0.001
acf_fcd (mean (SD))	0.02 (0.22)	-0.04 (0.20)	p<0.001	p<0.001
lnVAR_rcl (mean (SD))	1.23 (0.34)	1.33 (0.69)	0.005	0.022
lnVAR_huo (mean (SD))	2.01 (0.49)	1.74 (0.40)	p<0.001	p<0.001
acf_webfb (mean (SD))	1.29 (0.55)	1.20 (0.64)	p<0.001	0.036
acf_last (mean (SD))	0.10 (0.24)	0.08 (0.21)	0.046	0.035
lnVAR_ebfb (mean (SD))	1.64 (1.05)	1.31 (0.68)	p<0.001	p<0.001
acf_rcl (mean (SD))	0.02 (0.23)	-0.02 (0.29)	p<0.001	p<0.001
acf_ebzc (mean (SD))	-0.06 (0.18)	0.01 (0.22)	p<0.001	p<0.001
**GLM: 28d of a second-order polynomial quantile regression with a median 0.75**				
lnVAR_huo (mean (SD))	2.04 (0.62)	1.81 (0.46)	p<0.001	p<0.001
lnVAR_rfcp (mean (SD))	0.91 (0.32)	0.94 (0.35)	0.006	0.048
lnVAR_ebzc (mean (SD))	1.13 (0.48)	0.96 (0.41)	p<0.001	p<0.001
lnVAR_emax (mean (SD))	1.47 (0.62)	1.49 (0.42)	0.013	0.048
acf_rcl (mean (SD))	0.10 (0.19)	0.08 (0.27)	p<0.001	p<0.001
acf_ebfb (mean (SD))	0.07 (0.11)	0.07 (0.20)	0.05	0.013
acf_ebzc (mean (SD))	-0.02 (0.20)	0.09 (0.18)	p<0.001	p<0.001

Table S2. Abbreviation and full names for features in the analysis of the PLS model.

Abbreviation	Features	Abbreviation	Features
lnVAR_huo	LnVAR (log-transformed variance of deviations) of daily activity	lnVAR_rfl	lnVAR (log-transformed variance of deviations) of daily rumination time
lnVAR_max	lnVAR (log-transformed variance of deviations) of peak value of electricity conductivity	lnVAR_rcl	lnVAR (log-transformed variance of deviations) of daily milk yield
lnVAR_ebzc	lnVAR (log-transformed variance of deviations) of standard deviation change of conductivity	lnVAR_ebfb	lnVAR (log-transformed variance of deviations) of daily percentage change in the electrical conductivity of milk
lnVAR_emax	lnVAR (log-transformed variance of deviations) of standard deviation of maximum conductivity change in last three shifts	lnVAR_webfb	lnVAR (log-transformed variance of deviations) of the sum of absolute values of the weighted percentage change in the electrical conductivity of milk
lnVAR_rfcp	lnVAR (log-transformed variance of deviations) of rumination deviation per 2 h	acf_ebzc	acf (autocorrelation value) of standard deviation change of conductivity
acf_webfb	acf (autocorrelation value) of the sum of absolute values of the weighted percentage change in the electrical conductivity of milk	acf_rcl	acf (autocorrelation value) of daily milk yield
acf_last	acf (autocorrelation value) of standard deviation of maximum conductivity change in last three shifts	acf_emax	acf (autocorrelation value) of peak value of electricity conductivity
acf_rfc	acf (autocorrelation value) (autocorrelation value) of daily rumination time	acf_ebzc	acf (autocorrelation value) of standard deviation change of conductivity
acf_ebfb	acf (autocorrelation value) of daily percentage change in the electrical conductivity of milk	acf_huo	acf (autocorrelation value) of daily activity
InVAR_rfcp	lnVAR (log-transformed variance of deviations) of daily rumination deviation per 2 h	acf_last	acf (autocorrelation value) of standard deviation of maximum conductivity change in the last three shifts
